# Community Structure in Methanogenic Enrichments Provides Insight into Syntrophic Interactions in Hydrocarbon-Impacted Environments

**DOI:** 10.3389/fmicb.2016.00562

**Published:** 2016-04-22

**Authors:** S. Jane Fowler, Courtney R. A. Toth, Lisa M. Gieg

**Affiliations:** Petroleum Microbiology Research Group, Department of Biological Sciences, University of Calgary, CalgaryAB, Canada

**Keywords:** methanogenesis, hydrocarbon biodegradation, syntrophy, microbial community composition, co-occurrence network analysis

## Abstract

The methanogenic biodegradation of crude oil involves the conversion of hydrocarbons to methanogenic substrates by syntrophic bacteria and subsequent methane production by methanogens. Assessing the metabolic roles played by various microbial species in syntrophic communities remains a challenge, but such information has important implications for bioremediation and microbial enhanced energy recovery technologies. Many factors such as changing environmental conditions or substrate variations can influence the composition and biodegradation capabilities of syntrophic microbial communities in hydrocarbon-impacted environments. In this study, a methanogenic crude oil-degrading enrichment culture was successively transferred onto the single long chain fatty acids palmitate or stearate followed by their parent alkanes, hexadecane or octadecane, respectively, in order to assess the impact of different substrates on microbial community composition and retention of hydrocarbon biodegradation genes. 16S rRNA gene sequencing showed that a reduction in substrate diversity resulted in a corresponding loss of microbial diversity, but that hydrocarbon biodegradation genes (such as *assA/masD* encoding alkylsuccinate synthase) could be retained within a community even in the absence of hydrocarbon substrates. Despite substrate-related diversity changes, all communities were dominated by hydrogenotrophic and acetotrophic methanogens along with bacteria including *Clostridium* sp., members of the Deltaproteobacteria, and a number of other phyla. Microbial co-occurrence network analysis revealed a dense network of interactions amongst syntrophic bacteria and methanogens that were maintained despite changes in the substrates for methanogenesis. Our results reveal the effect of substrate diversity loss on microbial community diversity, indicate that many syntrophic interactions are stable over time despite changes in substrate pressure, and show that syntrophic interactions amongst bacteria themselves are as important as interactions between bacteria and methanogens in complex methanogenic communities.

## Introduction

Since the dawn of the industrial age, widespread use and processing of petroleum products has led to an increase in the hydrocarbon contamination of a wide range of environments. Despite increasing environmental awareness and improved remediation technologies, contamination of the subsurface with hydrocarbon mixtures remains a problem, as the fate of hydrocarbons in the subsurface is not fully understood especially under anoxic conditions. The exposure of subsurface environments to heavy organic loads such as hydrocarbons leads to the rapid development of anoxic conditions in which the majority of hydrocarbon biodegradation is thought to proceed via methanogenesis (Jones et al., 2008). This process is also important in many fossil energy reservoirs, wherein hydrocarbon metabolism over geologic time has led to the accumulation of biogenic methane in gas caps overlying oil legs (Jones et al., 2008). Many studies have now demonstrated that diverse hydrocarbon substrates can be biodegraded under methanogenic conditions (e.g., as reviewed in Foght, 2008; Gray et al., 2010; Gieg et al., 2014).

Methanogenic hydrocarbon metabolism requires the presence of at least two groups of organisms in order to proceed in a thermodynamically favorable manner: the syntrophic bacteria that catalyze the activation and subsequent degradation of hydrocarbons to methanogenic substrates (e.g., acetate, formate, CO_2_, and H_2_), and methanogenic archaea that bioconvert these simpler substrates to CH_4_ (plus CO_2_ or H_2_O). Methanogenic communities degrading hydrocarbon mixtures are typically diverse (Gray et al., 2010; An et al., 2013; Tan et al., 2015a), but how these microorganisms coordinate their metabolisms to utilize diverse hydrocarbons as carbon and energy sources and to conserve sufficient energy to support life is poorly understood (Gieg et al., 2014). Furthermore, the mechanisms involved in hydrocarbon activation are not fully understood, though fumarate addition has emerged as a key mechanism for the activation of aliphatic, substituted monoaromatic hydrocarbons, and substituted polycyclic aromatic hydrocarbons under various anaerobic electron-accepting conditions (Foght, 2008; Widdel and Musat, 2010; Callaghan, 2013). Alkylsuccinate synthase (encoded by the *assA*/*masD* gene; *assA* will be the designate name used in this study) is the key enzyme responsible for addition of alkanes to fumarate (Callaghan et al., 2008; Grundmann et al., 2008), while benzylsuccinate synthase (*bssA*) adds fumarate to substituted aromatic hydrocarbons (Heider, 2007).

In this study, we describe four new methanogenic enrichment cultures that were used to assess community changes as a result of decreased substrate diversity and that were probed for the presence of fumarate addition genes. Two cultures degrading the long-chain fatty acids (LCFA) palmitate and stearate were established from a whole crude oil-degrading methanogenic culture (Gieg et al., 2008) as the inoculum. The LCFA-degrading cultures were subsequently transferred to their parent alkanes, hexadecane and octadecane, in order to see if these cultures maintained the ability to degrade the hydrocarbon substrates present in the original oil degrading culture after long-term incubation on LCFA. All of these cultures (including the whole crude oil-degrading culture) were subjected to pyrotag sequencing of the 16S rRNA gene. We hypothesized that variations in the microbial community composition would be related to the specific carbon substrate supplied, which could provide clues to the identity of hydrocarbon-degraders in the cultures. We expected that the crude oil-degrading culture, which is exposed to a diverse hydrocarbon mixture and is the original parent culture, would be the most biodiverse of the cultures. LCFA- and *n*-alkane-amended cultures were expected to exhibit less diversity due to the restriction of carbon and energy sources within the culture, and the dilution effects of successive transfers. We further postulated that community members that were maintained across the majority of cultures over time likely play fundamental roles in the syntrophic degradation of shared metabolic products such as fatty acids, acetate, and formate. In light of this, we conducted a co-occurrence network analysis including community members that were retained across the different cultures in an attempt to establish an understanding of the syntrophic interactions occurring in the cultures.

A better understanding of methanogenic hydrocarbon metabolism could lead to the improvement of biotechnological applications for *in situ* bioremediation and for the bioconversion of residual oil to methane as a tertiary energy recovery strategy from fossil-energy reservoirs. Furthermore, insight into the syntrophic lifestyle can help shed light on novel mechanisms for interspecies communication or coordination, interspecies electron and metabolite transfer, and energy conservation in low energy-yielding environments.

## Materials and Methods

### Culture Incubations

The inoculum for the cultures described herein was initially derived from gas condensate-contaminated aquifer sediments that were found to biodegrade whole crude oil under methanogenic conditions (Townsend et al., 2003). This original culture was subsequently amended with crude oil-containing crushed sandstone reservoir core material as previously described (Gieg et al., 2008) and was found to utilize *n*-alkanes (C_12_–C_29_) in whole crude oil; the culture is referred to herein as the residual oil culture. Based on previous reports suggesting relationships between the degradation of *n*-alkanes and their corresponding fatty acids (e.g., Aeckersberg et al., 1998), we hypothesized that the residual oil culture would have the ability to utilize LCFA. Thus, in 2008 the residual oil culture was used to establish new enrichments amended with palmitate or stearate. These LCFA were selected because they represented the corresponding fatty acids to *n*-alkanes (C_16_ and C_18_) in the mid-range of the alkane fraction biodegraded by the residual oil culture (Gieg et al., 2008). Initial incubations showed that palmitate and stearate were metabolized based on the visual disappearance of the waxy substrates, and increased methane production relative to controls (not shown). Since then, these LCFA-degrading enrichments have undergone repeated substrate amendment with 30 μmol of stearate or palmitate as needed and had been transferred three times since their establishment (30–50% v/v transfer) prior to conducting the work described herein. The hexadecane- and octadecane-amended cultures were subsequently established in 2011 from these LCFA-degrading cultures by inoculating substrate-depleted palmitate- and stearate-degrading cultures with 0.03 g (133 μmol) hexadecane (added neat) or 0.03 g (118 μmol) octadecane, dissolved in 2,2,4,4,6,8,8-heptamethylnonane (HMN; 0.5 g/mL). The reason for the use of HMN, an inert hydrocarbon carrier, is that octadecane is a solid at room temperature (unlike hexadecane), and was thus difficult to amend to sealed serum bottles without first being dissolved in a solvent. These *n*-alkane-amended cultures underwent a single transfer to new medium (50% v/v) and substrate amendment as described above prior to the analyses described here. All cultures were established and maintained in glass serum bottles containing anoxically prepared bicarbonate-buffered minimal salts freshwater medium with 0.01% resazurin as a redox indicator and 2.5% v/v cysteine sulfide as the reductant (Fowler et al., 2012). Incubations were sealed with butyl rubber stoppers and aluminum crimps, and contained a CO_2_/N_2_ (20/80 vol%) headspace. All substrate-amended incubations were established at least in triplicate. Parallel substrate-unamended and sterile substrate-containing controls were also established.

### Methane Measurement

Methane was routinely monitored in all cultures and controls (as a surrogate for substrate utilization) using a Hewlett-Packard model 5890 Series gas chromatograph (GC) equipped with a flame ionization detector (200°C) with helium as the carrier gas. Headspace gas was sampled using a sterile 1 mL syringe flushed with 10% CO_2_ in N_2_ (Fowler et al., 2012). Injections were carried out at 150°C onto a packed stainless steel column (18″ long × 1/8″ i.d., Poropak R 80/100, Supelco) held isothermally at 100°C.

### DNA Extraction, PCR, and Pyrotag Sequencing

DNA was extracted using a modified phenol–chloroform method with bead beating. Cells (6 mL total) were repeatedly centrifuged at 18 000 × *g* for 10 min to pellet cells in 2 mL bead beating tubes containing 0.3 g of 0.01 mm and 0.1 g of 0.5 mm zirconia/silica beads (BioSpec Products). Cells were resuspended in 300 μL of lysis buffer (500 mM Tris, 100 mM NaCl, 10% SDS, pH 8) and 300 μL of chloroform-isoamyl alcohol (24:1). Bead beating was carried out at 6.0 m/s for 45 s. DNA was extracted by phenol chloroform-isoamyl alcohol extraction followed by RNAse and proteinase K treatment and a final phenol then chloroform-isoamyl alcohol extraction step. DNA was precipitated in sodium acetate (3 M, pH 7) and cold 100% ethanol at 18 000 × *g* for 20 min. Pellets were washed with cold 70% ethanol and resuspended in nuclease-free water (Fowler et al., 2012). Genomic DNA was found to be within the range of 1.41–9.49 ng/μL using Qubit fluorometry (Invitrogen).

Pyrotag sequencing was carried out following a two-step PCR method. In the first PCR step, DNA was amplified using universal primers 926F (AAACTYAAKGAATTGACGG) and 1392R (ACGGGCGGTGTGTRC) targeting the V6, V7, and V8 regions of the 16S rRNA gene in 25 μL reactions containing 2x PCR Master Mix (Fermentas), 0.2 μM of each primer and 1 μL of DNA using the following thermocycling protocol: 95°C 3 min; 25 cycles of 95°C (30 s), 55.0°C (45 s), 72.0°C (90 s); final extension 72°C 10 min. In order to attach barcode and adaptor sequences for 454 multiplex sequencing, a secondary PCR was carried out with primers 454T-FB-926F (which included the 25 bp B-adaptor sequence CTATGCGCCTTGCCAGCCCGCTCAG 5′ to the primer sequence) and 454T-FA-1392R (which included the 25 nt A-adaptor sequence CGTATCGCCTCCCTCGCGCATCAG and a variable 10 nt barcode sequence 5′ to the primer sequence). Reactions were prepared as above with modified thermocycling conditions: 95°C 3 min; 10 cycles of 95°C (30 s), 55.0°C (45 s), 72.0°C (90 s); final extension 72°C 10 min.

All PCR products containing adaptors and barcodes were then purified using a commercially available kit (Qiagen PCR Purification Kit). Amplicons were quantified by Qubit fluorometry (Invitrogen) according to the manufacturer’s protocol and were sequenced by 454 sequencing at the McGill University and Genome Quebec Innovation Centre (Montreal, Canada) using a GS FLX Titanium Series Kit XLR70 (Roche). Microbial community sequencing data are available in GenBank under the accession numbers SRR090429–SRR090434 for the residual oil and palmitate/stearate-degrading enrichments and under SRX1585942 (C_16_) and SRX1585943 (C_18_) for the *n*-alkane-amended enrichments.

### Bioinformatics Analysis

Analysis of 16S rRNA gene pyrotag data was carried out using the Phoenix 2 pipeline (Soh et al., 2013). Briefly, quality control was performed to remove low quality and chimeric sequences. Dereplication was performed (99% identity threshold) and sequences were clustered into OTUs at 3% distance using the average linkage algorithm. OTUs were mapped to taxa using the RDP classification algorithm within the SILVA training dataset (Pruesse et al., 2007). Biodiversity and other statistical measures were generated using mothur commands (Soh et al., 2013). In the results shown here, taxa comprising a single read were excluded from analysis as singleton and rare OTUs can be the result of sequencing errors, and rare taxa were not the focus of this analysis. Within Phoenix 2, Pearson correlations were calculated among shared OTUs within the five cultures. Microbial co-occurrence networks were constructed and visualized in Cytoscape v3.2.1 (Shannon et al., 2003) with OTUs that occurred in a minimum of three of five cultures with a minimum total abundance of 50 reads, and a Pearson correlation of at least 0.75.

### Alkylsuccinate Synthase Gene Amplification and Sequencing

Eight established alkylsuccinate synthase (*assA*) and five benzylsuccinate synthase (*bssA*) gene primers, and their respective reaction thermocycling conditions, were used to probe extracted DNA for the presence of fumarate addition enzymes (Washer and Edwards, 2007; Callaghan et al., 2010). PCR reactions were prepared with 12.5 μL 2x PCR Master Mix (Fermentas), 9 μL RNase free water, 0.5 μL each of a forward gene primer and corresponding reverse primer (10 μM), and 1 μL of template DNA. Amplicons of expected size were verified on a 1% agarose gel and subsequently purified using the QiaQuick PCR Purification Kit (Qiagen) according to the manufacturer’s protocol. The resulting purified amplicons were directly sequenced and queried against the NCBI non-redundant nucleotide database using BLASTN to identify homology to known sequences. A single *assA* gene fragment (523 bp; 393 bp after removing low quality nucleotide sequence ends) was amplified from each of the residual oil-, stearate-, and octadecane-amended enrichment cultures using primers 1432F and 1936R described by Callaghan et al. (2010).

Multiple alignments of the amplified sequences and representative sequences covering the same region were generated using the T-Coffee algorithm within the Centre for Genomic Regulation database (Notredame et al., 2000). Bootstrapped maximum likelihood trees (100 replicates) were constructed in MEGA6 (Tamura et al., 2013). A consensus tree was constructed using the Tamura–Nei model (Tamura and Nei, 1993) with complete deletions. Sequences for the *assA* genes reported in this study are available in GenBank under the accession numbers KU094062, KU094063, and KU094064.

## Results

### Methane Production from LCFA and Hydrocarbons

**FIGURE 1:**
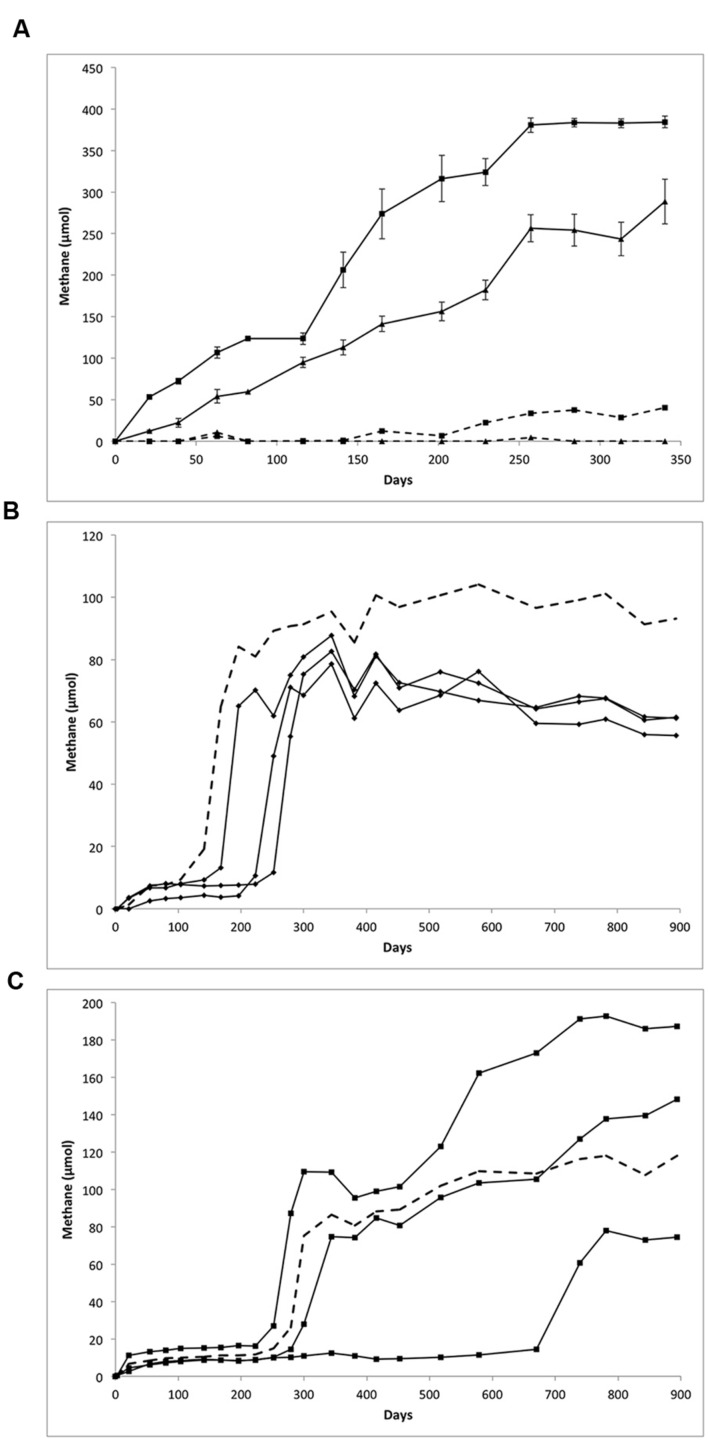
**Methane production from the biodegradation of **(A)** LCFAs, **(B)** hexadecane, and **(C)** octadecane.** **(A)** Methane production from palmitate (triangles) and stearate (squares) amended cultures over 340 days. The 30 μmol of palmitate or stearate was added on day 0, resulting in the production of 289 μmol of methane from palmitate (mean of five replicates) and 384 μmol of methane from stearate (mean of six replicates). Error bars show standard error. Dotted line represents substrate unamended control cultures. **(B)** Methane production from hexadecane amended culture over the course of 894 days; replicates were plotted individually (diamonds). Cultures were amended with 133 μmol of hexadecane (0.03 g) resulting in the production of an average of 60 μmol of methane. **(C)** Methane production from octadecane amended cultures over 894 days; replicates were plotted individually (squares). Cultures were amended with 118 μmol of octadecane on day 0 resulting in the production of methane ranging from 75 to 187 μmol. In **(B,C)**, methane production from unamended controls (dotted lines) may be related to the degradation of hydrocarbon substrate carried over during culture transfer or to the presence of cysteine added as a reductant.

Methane production was monitored following routine transfer and substrate amendment of the LCFA- and *n*-alkane-amended cultures, shown in **Figure 1**. Over the course of a 340-day incubation, the LCFA-degrading cultures appeared to completely consume their respective substrate based on a visual inspection wherein the waxy white substrate particles completely disappeared (relative to sterile controls). Based on the amounts of methane measured (**Figure 1A**) and the theoretical stoichiometric equations (Eqs. 1 and 2; Symons and Buswell, 1933) for the production of methane from 30 μmol of LCFAs, approximately 84 and 98% of palmitate or stearate, respectively, were metabolized via methanogenesis.

$$C_{16}H_{32}O_{2}+7H_{2}O\to 4.5CO_{2}+11.5CH_{4}\,\;\left(Pa\,\mathrm{lm}itate\right)$$

$$C_{18}H_{36}O_{2}+8H_{2}O\to 5CO_{2}+13CH_{4}\,\;\left(Stearate\right)$$

Similar amounts of methane were produced from these cultures over successive transfers (data not shown). These results are in line with previous work on methanogenic cultures, where about 64–98% of theoretically predicted methane is produced (assuming 100% conversion of substrates to methane). The balance of carbon presumably goes to the production of biomass, with a small amount being lost during headspace sampling and due to adsorption to the stopper (Stadtman and Barker, 1951; Zengler et al., 1999; Fowler et al., 2012). Substrate-unamended cultures produced 0 μmol (palmitate) and 41 μmol (stearate) methane.

The primary transfers of the *n*-alkane amended cultures produced variable amounts of methane in different replicates (**Figures 1B,C**). A long lag period was observed prior to methane production ranging from 141 days up to almost 600 days for one octadecane-amended enrichment. Methane production in the hexadecane-amended replicates was fairly uniform after 894 days of incubation, yielding approximately 60 μmol CH_4_ (**Figure 1B**). Methane production in the octadecane-degrading cultures was more varied; 187, 148, or 75 μmol CH_4_ were produced over 894 days from each of the three replicates (**Figure 1C**).

Methane production in the corresponding unamended controls incubated alongside the *n*-alkane-amended cultures was substantial (93.1 μmol and 118.0 μmol). This effect may be related to carryover of any undegraded hydrocarbon substrate when the cultures underwent primary transfer. However, a possible alternative carbon source for methane production is the cysteine present in the 2.5% cysteine sulfide added to these cultures as a reductant, which has been shown to be metabolized in other methanogenic hydrocarbon-degrading enrichment cultures (Toth, unpublished results). Based on the theoretical stoichiometric conversion of cysteine to methane (4C_3_H_7_NO_2_S + 6 H_2_O + 4H^+^ → 4H_2_S + 4NH_4_^+^+ 5CH_4_ + 7CO_2_), the amount of cysteine sulfide added as a reductant could result in the production of up to 107 μmol methane in each of the cultures. Given these calculations, methane production from palmitate-, stearate-, and octadecane-amended cultures exceeded the methane expected from cysteine sulfide alone, strongly suggesting that the LCFAs or octadecane are serving as the substrates for methane production. Methane production in the hexadecane-amended cultures, however, did not exceed 107 μmol, thus it cannot be concluded that hexadecane served as a substrate for methane production in this culture.

### Microbial Community Dynamics in Methanogenic Cultures

Pyrotag sequencing of the 16S rRNA gene was carried out to examine and compare the microbial communities in each of the cultures (**Table 1**). The residual oil culture, which served as the inoculum source for the palmitate and stearate cultures, was included in this analysis to determine whether or how transfers onto single carbon sources impact the microbial community composition. Quality controlled reads were clustered into OTUs at a 3% distance. Rarefaction analysis revealed that at a clustering distance of 3%, none of the samples were sequenced to saturation, however, at a clustering distance of 5%, the samples were approaching saturation (**Supplementary Figure S1**, top). The number of OTUs observed in each of the cultures (3% distance) varied considerably. The residual oil culture harbored the greatest number of OTUs, and observed OTU numbers decreased following the order in which the cultures were successively enriched [e.g., residual oil (297) → LCFA (151/164) →*n*-alkane (114/120); **Table 1**]. The Chao index, which estimates the actual number of OTUs in each sample, also indicated that the residual oil culture was the most diverse, and that diversity decreased with each enrichment step. As expected from the observed OTUs and Chao estimates, as well as the diversity of hydrocarbon substrates and initial culture for enrichment, the Shannon and Simpson diversity indices also indicated that the residual oil culture was the most diverse of the cultures (**Table 1**). The octadecane-amended culture also had high Shannon and low Simpson values, despite comprising lower observed OTUs and Chao values relative to the LCFA and hexadecane-amended cultures. This is due to greater evenness in this culture (**Table 2**). As expected, Bray–Curtis dissimilarity analysis revealed that the microbial communities of the hexadecane- and octadecane-amended cultures were most closely related to each other, and the stearate- and palmitate-degrading cultures were also closely related to one another, while the residual oil degrading culture was the most distantly related culture (**Supplementary Figure S1**, bottom).

**Table 1:** Features of 16S rDNA pyrosequencing and alpha diversity statistics based on analysis at 0.03 distance for all methanogenic cultures analyzed in this study.

Feature/Culture	Residual oil	Palmitate	Stearate	Hexadecane	Octadecane
Reads (pre/post QC)	13381/7621	8294/4806	9501/6051	21575/19202	10200/8699
#OTUs observed	297	151	164	114	120
Shannon	3.15	1.92	1.96	1.82	2.28
Simpson	0.12	0.31	0.37	0.28	0.21
Chao	817	313	292	197	185


**Table 2:** Relative abundance (%) of most abundant taxa in methanogenic cultures as determined by 16S rRNA gene pyrosequencing.

Taxon	Residual oil	Palmitate	Stearate	Hexadecane	Octadecane
*Clostridium*	30.4	52.1	60.4	43.0	42.0
*Methanoculleus*	3.2	1.0	2.5	6.1	15.4
*Methanolinea*	3.5	0	0.2	0	13.3
WCHA1-57	0.2	0.022	0.3	3.5	6.9
*Methanosaeta*	12.0	8.0	10.9	31.3	6.3
*Desulfovibrio*	3.7	0.6	0.9	1.0	2.3
*Candidatus Methanoregula*	0.7	12.9	1.2	8.5	2.2
*Ruminococcaceae*	0.2	0.7	1.3	0.9	2.1
*Anaerolineaceae* uncultured	5.7	2.0	8.7	1.4	1.8
*Synergistaceae* uncultured	0.03	0.23	0.3	0.5	1.6
*Geobacter*	0.4	0.1	0.3	1.2	0.8
*Spirochaetaceae* uncultured	2.2	0.6	1.1	0.1	0.6
*Lachnospiraceae* Incertae Sedis	0.0	1.1	4.4	0.08	0.2
*Smithella*	16.1	15.4	2.0	0.05	0.3
*Anaerobacter*	1.0	1.2	1.2	0.3	0.20
*Thermosinus*	0.04	0.4	1.0	0.005	0.01
*Enterobacter*	3.4	0	0	0	0
vadinHA17	1.6	0	0	0.9	0.1
*Sedimentibacter*	1.4	0.8	0.3	0.06	0.09
*Proteiniphilum*	1.2	0.4	0.4	0.1	0.2
**Total**	**87.0**	**97.4**	**97.1**	**99.0**	**96.5**


**FIGURE 2:**
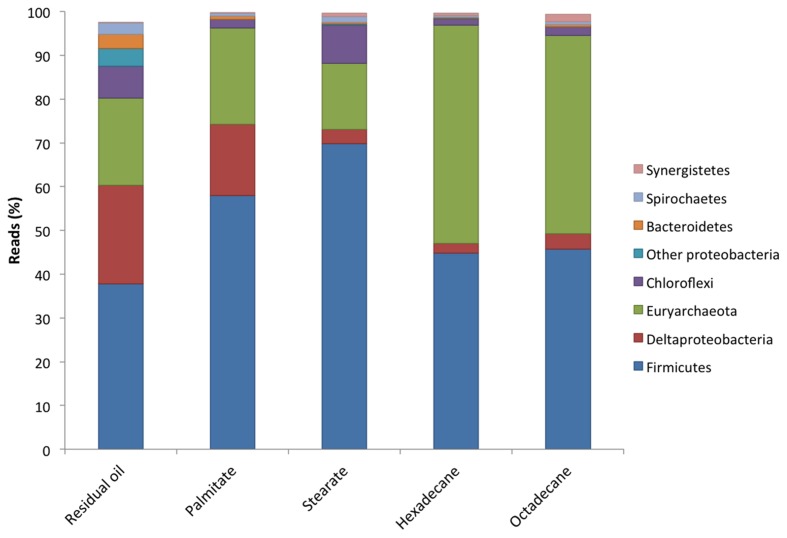
**Microbial community compositions at the phylum level of the methanogenic enrichment cultures based on 16S rRNA gene pyrosequencing**.

All five cultures were dominated by members of the Firmicutes making up between 37.8 and 69.8% of the sequence reads from each culture (**Figure 2**). The highest abundances of Firmicutes were found in the LCFA-degrading cultures, with 57.9 and 69.8% in the palmitate- and stearate-degrading cultures, respectively (**Figure 2**). Other dominant phyla included the Euryarchaeota and Deltaproteobacteria. Together, members of these three phyla comprised at least 80.2% (residual oil) and up to 96.8% (hexadecane) of each of the microbial communities (**Figure 2**). Euryarchaeota (methanogens) were particularly abundant in the hexadecane- and octadecane- amended cultures where they made up 49.8 and 45.4% of the reads, respectively. Aside from the residual oil culture that exhibited the highest richness and evenness, the only other phylum that made up greater than 1% of the community in any culture was Chloroflexi (**Figure 2**). In all cultures, the Chloroflexi were dominated by a single OTU affiliating with *Anaerolineaceae*. At lower taxonomic levels, *Clostridium* sp. was the most abundant genus in all cultures, comprising between 30.4 and 60.4% of the sequence reads for each culture (**Table 2**). In the residual oil culture, the next most abundant organism was *Smithella* sp. (16.1%). Though *Smithella* sp. was also abundant in the palmitate-amended culture (15.4%) it was far less abundant in the stearate-, octadecane-, and hexadecane-amended cultures (**Table 2**). Several genera of methanogenic archaea also comprised considerable fractions of the microbial communities, particularly in the *n*-alkane-amended cultures. These included hydrogenotrophic (*Methanoculleus*, *Methanolinea*, and *Methanoregula*) and acetotrophic (*Methanosaeta*) methanogens. Interestingly, the acetotrophic methanogen *Methanosaeta* was the most abundant type of methanogen in the residual oil-, palmitate-, stearate-, and hexadecane-amended enrichments, while hydrogenotrophic methanogens *Methanoculleus* and *Methanolinea* dominated in the octadecane-amended enrichment. A methylotrophic methanogen was also detected at comparatively lower abundance in all cultures (*Methanomethylovorans*), but was most abundant in the *n*-alkane-amended cultures (0.14–0.62% of hexadecane and octadecane communities, respectively). In addition, the archaeon WCHA1-57 (possibly a novel lineage of methanogens, Saito et al., 2015) was also particularly abundant in the octadecane degrading culture (**Table 2**).

### Microbial Co-occurrence Network Analysis

**FIGURE 3:**
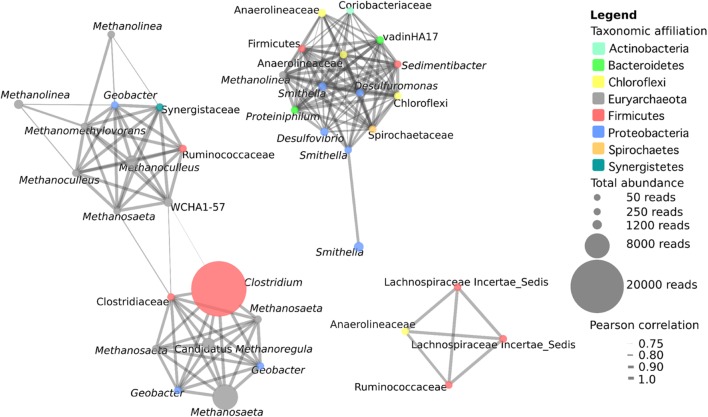
**Microbial co-occurrence network from five methanogenic enrichment cultures.** OTUs present in a minimum of three cultures at a minimum abundance of 50 reads with a positive Pearson correlation of at least 0.75 were included in the analysis. The size of a node represents the abundance of the OTU across the five samples, and the width of the edges represents the strength of the correlation.

Microbial co-occurrence analysis was conducted with OTUs that were present in at least three samples with a total minimum abundance of 50 reads and a positive Pearson correlation greater than 0.75. This included 37 OTUs, with a total of 155 interactions. Co-occurrence analysis revealed the presence of three distinct networks within the samples (**Figure 3**). The first network contains 18 OTUs, and consists primarily of hydrogenotrophic (*Methanoculleus*, two OTUs; *Methanolinea*, two OTUs; *Methanoregula*, one OTU), acetotrophic (*Methanosaeta*, four OTUs) and methylotrophic (*Methanomethylovorans*, one OTU) methanogenic archaea and one OTU related to the archaeon WCHA1-57 of the *Thermoplasmata*. This network also includes seven syntrophic bacteria including a highly abundant OTU corresponding to *Clostridium* sp. as well as a *Clostridiaceae*, *Geobacter* spp. (three OTUs), and OTUs most closely related to members of the *Synergistaceae* and *Ruminococcaceae*. Some of the strongest interactions in this network occur between methanogens and syntrophic bacteria, likely related to the transfer of hydrogen or formate, acetate and/or electrons from the syntroph to the methanogen. However, there are also interactions between methanogenic OTUs with particularly strong correlations between an OTU related to *Methanomethylovorans* with two *Methanoculleus* OTUs. Interestingly, this network actually consists of two interconnected networks linked by the two *Clostridium/Clostridiaceae* OTUs, suggesting a central role for *Clostridiaceae* in the interaction with methanogens in these cultures.

The second network consists primarily of syntrophic bacteria from diverse phyla, as well as a single methanogen OTU affiliating with the hydrogenotroph *Methanolinea* (**Figure 3**). The 14 bacterial OTUs identified are members of the Deltaproteo bacteria (five OTUs), Chloroflexi (three OTUs), Firmicutes (two OTUs), Bacteroidetes (two OTUs), Actinobacteria (one OTU), and Spirochaetes (one OTU). With the exception of one *Smithella* OTU, which is only connected to one other *Smithella* OTU, there is dense and strong connectivity within this network and all other OTUs have between 9 and 13 connections. The number and the strength of the interactions within this network suggest that these OTUs play central and cooperative roles in the degradation of hydrocarbon or LCFA substrates in these cultures. The third network consists of four bacterial OTUs comprising three OTUs affiliated with *Clostridiales* (two uncultured *Lachnospiraceae* OTUs, and one *Ruminococcaceae*), as well as one OTU affiliated with *Anaerolineaceae* (Chloroflexi). The organisms in this network were not highly abundant (60–277 reads) but all of the OTUs were found to strongly co-occur with one another, indicating the presence of a second bacterial cooperative metabolic network, this time with the complete absence of methanogens (**Figure 3**).

### Detection of Fumarate Addition Genes

Established alkylsuccinate synthase (*assA*) and benzylsuccinate synthase (*bssA*) primer sets were used to probe extracted metagenomic DNA from all cultures (including the residual oil culture) for the presence of fumarate addition genes (Washer and Edwards, 2007; Callaghan et al., 2010). A single *assA* gene fragment was amplified from each of the residual oil-, stearate-, and octadecane-amended enrichment cultures. There was 99.4% nucleotide sequence identity among the gene fragments, suggesting that all three amplicons belong to the same species. These results show that alkane biodegradation potential (via fumarate addition) was maintained across culture transfers despite changes in carbon substrate. Notably, the *assA* gene was undetectable in palmitate- and hexadecane-enriched microcosms; these findings substantiate the observation that hexadecane did not appear to be metabolized to methane in this study (**Figure 1B**). Further, *bssA* could not be detected in any sample with the evaluated primer sets.

**FIGURE 4:**
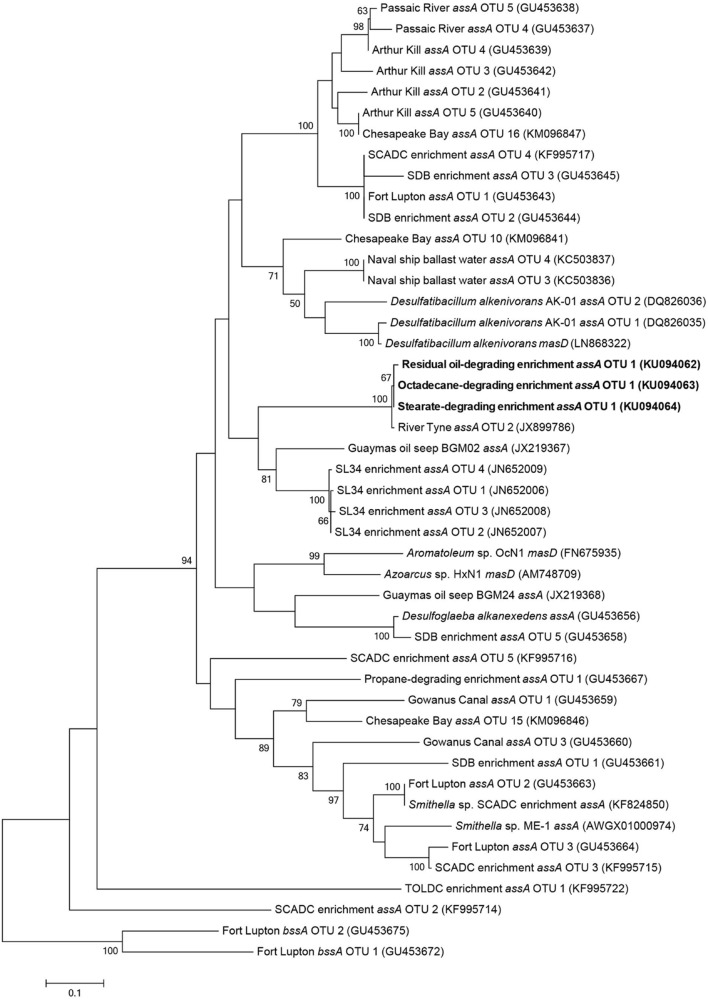
**Maximum likelihood tree showing the affiliation of *assA* gene fragments (**this study, bold**) with previously published reference strains, enrichment cultures, and environmental samples.** Evolutionary analyses of aligned nucleotide sequences (320 bp) were conducted in MEGA6 (Tamura et al., 2013). The consensus tree was constructed using the Tamura–Nei model (Tamura and Nei, 1993) with complete deletions (for a total of 288 positions in the final dataset) and performing 100 bootstrap replicates. Bootstrap values below 50% are not shown. Benzylsuccinate synthase (*bssA*) genes cloned from gas condensate-contaminated sediments near Fort Lupton, CO, USA (Callaghan et al., 2010) were used to root the tree.

Maximum likelihood trees of *assA* gene fragments revealed that sequenced amplicons were most closely related to three identical uncultured prokaryote clones (99% sequence similarity) isolated from sulfate-reducing, alkane-degrading River Tyne sediments (Sherry et al., 2013) (**Figure 4**). The *assA* gene fragments also clustered closely to those retrieved from other anaerobic long chain alkane-degrading enrichment cultures (C_15_–C_20_; SL34 enrichment OTUs, Mbadinga et al., 2012) and an oil seep (von Netzer et al., 2013).

## Discussion

Reports of methane generation from *n*-alkanes, and the description of the communities mediating these transformations have become increasingly widespread in recent years (e.g., Zengler et al., 1999; Gieg et al., 2008; Jones et al., 2008; Callaghan et al., 2010; Sherry et al., 2013; Berdugo-Clavijo and Gieg, 2014; Abu Laban et al., 2015; Bian et al., 2015; Tan et al., 2015b). Alkanes comprise an abundant fraction of many crude oils, thus their biodegradation under anaerobic conditions is of practical relevance to biotechnological applications in fossil energy reservoirs and fuel-contaminated sites. There remains much to be learned with regards to the pathways, enzymes, and genes involved in strictly anaerobic alkane degradation, as well as the organisms and interactions amongst organisms that methanogenically metabolize hydrocarbons.

In this study, we describe new methanogenic LCFA- and *n*-alkane-degrading cultures, including analysis of their community structure and amplification of known biodegradative genes. Co-occurrence network analysis of the microbial communities of the five related cultures was conducted in order to make a first step in unraveling syntrophic interactions in methanogenic hydrocarbon-degrading systems. As the downstream degradation of both alkanes and fatty acids proceed via a common pathway – β-oxidation, followed by conversion to methanogenic substrates and methane production (Callaghan, 2013) – syntrophic interactions are expected to be similar regardless of the hydrocarbon or fatty acid substrate being degraded. We propose that over time, stable and efficient syntrophic interactions have evolved within the microbial community and that these interactions are fairly resilient to the substrate being degraded.

Degradation of crude oil components was previously demonstrated by the source inoculum for the cultures described herein (Gieg et al., 2008). This culture was subsequently transferred to the LCFAs palmitate and stearate, and then these cultures were transferred to their respective parent alkanes, hexadecane and octadecane, to examine the effects of different substrates on microbial community structure and to determine whether the ability to degrade hydrocarbons was maintained following long-term incubation on LCFAs. Over several years of routine culture transfer and substrate amendment, the LCFA-degrading cultures typically converted approximately 84–98% of their fatty acid substrates to methane (plus CO_2_ or H_2_O; **Figure 1A**). Alkane cultures were subject to a single culture transfer, after which methane production was monitored for close to 900 days. After an extended lag period, methane generation from octadecane became apparent (**Figure 1C**), while the degradation of hexadecane could not be confirmed (**Figure 1B**) because the methane production did not exceed the maximum amount of methane that could be derived from the reductant, cysteine sulfide. Lag periods exceeding several weeks or months have been reported for other methanogenic hydrocarbon-degrading cultures (Edwards and Grbić-Galić, 1994; Townsend et al., 2003; Berdugo-Clavijo and Gieg, 2014). This delay may be related to a toxic effect as inhibition of microbes by hydrocarbon substrates has been well documented and is thought to be related to interference with biological membranes (Sikkema et al., 1995).

The methane production observations aligned with the results of fumarate addition gene amplification efforts. Alkylsuccinate synthase, the enzyme involved in anaerobic *n*-alkane activation via addition to fumarate, was previously detected in the sediments from which these cultures were initially derived (Callaghan et al., 2010). In the present study, we detected *assA* amplicons, all presumably derived from a single species, in the residual oil-, stearate-, and octadecane-amended cultures. These results show that the potential ability to biodegrade hydrocarbons can be retained within a syntrophic microbial community even following the long-term absence of hydrocarbon exposure. However, gene detection does not indicate actual expression, thus further studies will be required to confirm that this gene is actually expressed during biodegradation under these different substrate conditions. In contrast, the *assA* gene was not detected in the palmitate- or hexadecane-amended cultures (**Figure 4**), for reasons that are not clear given its detection in the stearate and octadecane enrichments. Palmitate metabolism does not require *assA*, thus a simple explanation is that the gene was lost (i.e., the species harboring this gene was lost) upon transfer from the residual oil culture to palmitate. This explains why a subsequent transfer of the palmitate-degrading culture onto hexadecane did not lead to the biodegradation of this *n*-alkane (**Figure 1B**). Another possibility is that the specific *assA*-containing organism in these cultures is involved in the degradation of longer chain alkane substrates, and was thus not capable of hexadecane degradation. If this were true, it would indicate a distinct difference between the fumarate addition genes involved in the degradation of octadecane and higher alkanes, and hexadecane and shorter alkanes. While the necessary evidence to fully test this hypothesis is not yet available due to a shortage of *assA* gene sequences with known substrate range, this idea was previously postulated for the *assA* genes involved in the degradation of short chain alkanes (*n*-C_3_-C_10_; Callaghan et al., 2010; Tan et al., 2015a). A distinction between hexadecane and octadecane would not be particularly surprising, as hexadecane is a liquid at ambient temperature, while octadecane is a solid, making the bioavailability of each different in an aqueous environment under mesophilic conditions. A similar hypothesis was made for gaseous alkane *assA* being phylogenetically distinct from non-gaseous alkane fumarate addition enzymes (Musat, 2015). Phylogenetic analysis in the present study of the amplified *assA* genes with known references and environmental samples did not reliably pinpoint the organism(s) harboring this *assA* gene. While the phylogeny of *bssA* (encoding the alpha subunit of benzylsuccinate synthase) is now generally well enough resolved to infer the clade involved in aromatics activation (von Netzer et al., 2013), this is not yet the case for *assA* (**Figure 4**). In our phylogenetic analysis, as in others (Callaghan et al., 2010), members of the Deltaproteobacteria (*Desulfoglaeba* sp.) grouped more closely with Betaproteobacteria alkane degraders (*Azoarcus* sp., *Aromatoleum* sp.), than with other Deltaproteobacteria (*Desulfatibacillum* sp.) which may indicate that genes encoding alkylsuccinate synthase are more closely related based on the specific alkane substrates being degraded rather than phylogeny or that they are subject to a high degree of horizontal gene transfer (this is currently unknown). It remains to be seen if the purification of additional strictly anaerobic alkane degraders, or the improved description of alkane degraders from the environment or enriched cultures will eventually result in the ability to predict either the taxonomic affiliation of alkane degraders based on phylogenetic analysis of the *assA* gene and/or the substrate range of the degraders. Members of the Deltaproteobacteria are often cited as key alkane and/or fatty acid degraders in methanogenic cultures (e.g., members of the *Syntrophaceae* such as *Syntrophus*/*Smithella* spp., Gray et al., 2011; Cheng et al., 2013; Embree et al., 2014; Tan et al., 2014; Mathai et al., 2015) along with other sulfate-reducing alkane degrading Deltaproteobacterial isolates (Cravo-Laureau et al., 2005; Davidova et al., 2006; Callaghan et al., 2008). While *Smithella* sp. was abundant (16%) in the residual oil culture, it was present at <2% abundance in the stearate- and octadecane-degrading cultures (**Table 2**) and the recovered *assA* gene fragments did not cluster with the *assA* of known *Smithella* sp. (**Figure 4**). These findings suggest that this taxon is not the main stearate- or octadecane-degrading organism in these enrichments. No Deltaproteobacterial OTUs previously associated with hydrocarbon biodegradation were particularly enriched in the octadecane-amended culture (**Table 2**), suggesting that an as of yet unidentified alternate organism(s) catalyzes the activation of this *n*-alkane. The *bssA* gene was not detected in any of the enrichments, which was expected, as the residual oil-amended microcosms contained negligible concentrations of substituted monoaromatic hydrocarbon substrates such as toluene (Gieg et al., 2008).

In comparing the microbial communities of the five different cultures, we found that the microbial richness observed was related to the order in which the cultures were enriched, with the most highly enriched (*n*-alkane-incubated) cultures containing the lowest species richness (**Table 1**). A much greater richness and evenness was observed in the presence of more diverse hydrocarbon substrates as found in the residual oil-containing culture (**Tables 1** and **2**). In addition, there were substrate-specific variations in the microbial communities, with a particular enrichment of methanogens in the *n*-alkane-incubated cultures (**Table 2**). While observed and predicted richness decreased with degree of enrichment, evenness actually increased in both of the alkane-incubated cultures relative to the LCFA-degrading cultures (**Table 1**). Nonetheless, all cultures shared a similar microbial community structure and were dominated by members of the Firmicutes (*Clostridium* sp.), Deltaproteobacteria, and Euryarchaeota (mainly hydrogenotrophic and acetotrophic methanogens; **Figure 2**, **Table 2**). These findings are consistent with the previous clone library analysis of the residual oil culture (Gieg et al., 2008), and also of a toluene-degrading methanogenic culture derived from the same contaminated sediments (Fowler et al., 2012). This is not particularly surprising as the majority of known syntrophic bacteria are members of the Firmicutes or Deltaproteobacteria (Sieber et al., 2012), and the methanogenic archaea are members of the Euryarchaeota. The extremely high abundance of *Clostridium* sp. is also consistent with the community from the toluene degrading enrichment from the same sediments in which 30.7% of the culture was found to consist of *Clostridium* sp. (Fowler et al., 2012). While *Clostridium* sp. is an abundant organism in these new enrichment cultures, it is possible that its extremely high abundance is partly an artifact of PCR as *Clostridium* spp. often have multiple copies of 16S rRNA genes with 14 copies having been observed in a single genome (Větrovský and Baldrian, 2013). The presence of a large *Clostridium* sp. OTU and several *Clostridiaceae* sp. OTUs in the network analysis, and their connectivity to methanogens in this analysis suggests that, despite their high abundance, this clade is not involved in hydrocarbon activation, but is involved in the downstream conversion of smaller molecules to methanogenic intermediates (**Figure 3**). This is also in agreement with the results from the aforementioned toluene-degrading culture, in which the highly abundant *Clostridium* sp. did not incorporate ^13^C label from toluene during a 7-day time course experiment (Fowler et al., 2014). While it can not be ruled out that *Clostridium* spp. might be directly involved in hydrocarbon activation, these results collectively point toward a general role for *Clostridium* sp./*Clostridiaceae* in the downstream degradation of hydrocarbons to methanogenic substrates in oil-associated environments, rather than directly activating hydrocarbons in these cultures.

Co-occurrence network analysis revealed the presence of three distinct networks within the cultures (**Figure 3**). The first consisted of diverse methanogenic archaea including hydrogenotrophs, acetotrophs, and methylotrophs. These methanogens were found to co-occur with syntrophic bacteria including *Geobacter* sp., *Ruminococcaceae*, *Synergistetes*, and in particular, two *Clostridiaceae*-affiliated OTUs with a high degree of connectivity that linked the two sections of this network. This network is likely reflecting a number of direct interactions between syntrophic bacteria and methanogenic archaea involving interspecies metabolite transfer, and possibly even direct interspecies electron transfer (DIET) between *Geobacter* sp. and methanogens (Rotaru et al., 2014). The second network consisted of 14 OTUs of diverse syntrophic bacteria and a single *Methanolinea* sp. OTU. The syntrophic bacteria within this network were densely interconnected with a high mean degree of connectivity. Whether these OTUs are all connected due to a highly interactive syntrophic network, or due to the presence of a small number of organisms that interact with a large number of partners (which has previously been observed in these densely connected networks; Berry and Widder, 2014) is unknown. However, this network indicates that it is not only interactions between syntrophic bacteria and methanogens that are important in these communities, but also that interactions amongst syntrophic bacteria are substantial. The third network also emphasizes the importance of interactions amongst syntrophic bacteria as it consists solely of four bacterial OTUs related to obligate anaerobic fermenters (three *Clostridiales*, one *Anaerolineaceae*) that all co-occur. *Lachnospiraceae* and *Ruminococcaceae* are common inhabitants of GI tracts, anaerobic digesters, and other methanogenic environments (Nelson et al., 2011; Biddle et al., 2013). *Anaerolineaceae* are also commonly found in syntrophic environments including the GI tract and anaerobic digestors, where they are typically characterized as secondary fermenters that sequentially degrade fatty acids and/or carbohydrates to methanogenic or other syntrophic intermediates, and are known to associate with hydrogenotrophic methanogens (Yamada et al., 2006; Biddle et al., 2013; St-Pierre and Wright, 2013). *Anaerolineaceae* have also been previously detected in high abundance in alkane-degrading cultures and anaerobic oil-impacted environments (Savage et al., 2010; Sherry et al., 2013; Liang et al., 2015) including methanogenic and non-methanogenic syntrophic hydrocarbon-degrading cultures (Kleinsteuber et al., 2012). Further, *Anaerolineaceae* have previously been postulated to be involved in alkane activation under methanogenic conditions (Sherry et al., 2013; Liang et al., 2015). Thus, an alternative possibility is that *Anaerolineaceae* is involved in alkane activation, and subsequent fatty acid degradation is catalyzed by the *Clostridiales* OTUs, though additional evidence to support *Anaerolineaceae* as alkane degraders in these cultures is currently lacking. Overall, network analysis indicates that there are strong interactions between syntrophic bacteria and methanogens, but the strongest and most abundant interactions we observed in these cultures occurred amongst the bacteria themselves. This suggests the existence of numerous cooperative interactions between groups of bacteria as well as between bacteria and methanogens within syntrophic methanogenic ecosystems. While the use of co-occurrence networks can provide clues as to how organisms interact in syntrophic cultures, they must also be interpreted with caution. Co-occurrence in this analysis merely indicates that the organisms were observed to co-occur repeatedly, but does not preclude the possibility that co-occurring organisms merely share similar niches within these enrichment cultures and do not interact. However, due to the difficulty in elucidating syntrophic interactions in mixed cultures, we believe that co-occurrence network analysis provides a method that can be used to predict syntrophic relationships in complex communities when applied to multiple related communities.

In summary, we demonstrated the methanogenic bio degradation of palmitate, stearate, and octadecane in cultures derived from a whole crude oil-degrading enrichment culture (Gieg et al., 2008). The fact that octadecane degradation occurred following 3 years of pre-incubation on a non-hydrocarbon substrate (stearate) showed that alkane degraders can persist in environments despite the absence of hydrocarbons. In addition, we described the microbial communities of each of these cultures and a hexadecane-amended culture, and observed an expected diversity reduction when whole crude oil-amended cultures were successively transferred onto single carbon substrates. Confirmation of syntrophic interactions between individual OTUs ultimately requires physiological evidence. However, given the complexity of methanogenic communities, and the difficulty in culturing syntrophic bacteria as individuals or in co-culture, applying microbial co-occurrence network analysis provides a means to predict microbial interactions, enabling insight into potential interspecies interactions and the microbial foodwebs that exist in complex communities. By examining microbial co-occurrence in these cultures, we were able to identify organisms that were insensitive to the carbon substrate being metabolized, and examine their degree of co-occurrence with other community members. While these co-occurrences likely do not all represent syntrophic interactions, this is a first step toward identifying organisms that form associations within this stable syntrophic community. Our analysis reveals not only stable interactions between syntrophs and methanogens, including possible DIET interactions, but also strong interactions amongst the syntrophic bacteria themselves. These findings emphasize the complex foodwebs existing in methanogenic communities. Furthermore, these predictions can provide preliminary evidence for further hypothesis testing using metagenomic and/or metatranscriptomic data and/or physiological investigations.

## Author Contributions

SF and LG conceived the research. LG established the original oil and LCFA cultures and SF transferred and maintained subsequent enrichments. SF conducted the methane measurements, the 16s rRNA gene sequencing, and microbial co-occurrence network analysis for all cultures. CT assayed for and interpreted the biodegradation gene results. All authors participated in writing the manuscript.

## Conflict of Interest Statement

The authors declare that the research was conducted in the absence of any commercial or financial relationships that could be construed as a potential conflict of interest.

## References

[B1] Abu Laban N. A. Dao A. Semple K. Foght J. (2015). Biodegradation of C7 and C8 iso-alkanes under methanogenic conditions. *Environ. Microbiol.* 17 4898–4915. 10.1111/1462-2920.12643 25331365

[B2] Aeckersberg F. Rainey F. A. Widdel F. (1998). Growth, natural relationships, cellular fatty acids and metabolic adaptation of sulfate-reducing bacteria that utilize long-chain alkanes under anoxic conditions. *Arch. Microbiol.* 170 361–369. 10.1007/s002030050654 9818355

[B3] An D. Brown D. Chatterjee I. Dong X. Ramos-Padron E. Wilson S. L. (2013). Microbial community and potential functional gene diversity involved in anaerobic hydrocarbon degradation and methanogenesis in an oil sands tailings pond. *Genome* 56 612–618. 10.1139/gen-2013-0083 24237342

[B4] Berdugo-Clavijo C. Gieg L. M. (2014). Conversion of crude oil to methane by a microbial consortium enriched from oil reservoir production waters. *Front. Microbiol.* 5:197 10.3389/fmicb.2014.00197 PMC4017130 24829563

[B5] Berry D. Widder S. (2014). Deciphering microbial interactions and detecting keystone species with co-occurrence networks. *Front. Microbiol.* 5:219 10.3389/fmicb.2014.00219 PMC4033041 24904535

[B6] Bian X.-Y. Mbadinga S. M. Liu Y.-F. Yang S.-Z. Liu J.-F. Ye R.-Q. (2015). Insights into the anaerobic biodegradation pathway of n-alkanes in oil reservoirs by detection of signature metabolites. *Sci. Rep.* 5 9801 10.1038/srep09801 PMC4429370 25966798

[B7] Biddle A. Stewart L. Blanchard J. Leschine S. (2013). Untangling the genetic basis of fibrolytic specialization by Lachnospiraceae and Ruminococcaceae in diverse gut communities. *Diversity* 5 627–640. 10.3390/d5030627

[B8] Callaghan A. V. (2013). Enzymes involved in the anaerobic oxidation of n-alkanes: from methane to long-chain paraffins. *Front. Microbiol.* 4:89 10.3389/fmicb.2013.00089 PMC3653055 23717304

[B9] Callaghan A. V. Davidova I. A. Savage-Ashlock K. Parisi V. A. Gieg L. M. Suflita J. M. (2010). Diversity of benzyl- and alkylsuccinate synthase genes in hydrocarbon-impacted environments and enrichment cultures. *Environ. Sci. Technol.* 44 7287–7294. 10.1021/es1002023 20504044

[B10] Callaghan A. V. Wawrik B. Ní Chadhain S. M. Young L. Y. Zylstra G. J. (2008). Anaerobic alkane-degrading strain AK-01 contains two alkylsuccinate synthase genes. *Biochem. Biophys. Res. Commun.* 366 142–148. 10.1016/j.bbrc.2007.11.094 18053803

[B11] Cheng L. Ding C. Li Q. He Q. Dai L. R. Zhang H. (2013). DNA-SIP reveals that Syntrophaceae play an important role in methanogenic hexadecane degradation. *PLoS ONE* 8:e66784 10.1371/journal.pone.0066784 PMC3698093 23840866

[B12] Cravo-Laureau C. Grossi V. Raphel D. Matheron R. Hirschlér-Rea A. (2005). Anaerobic n-alkane metabolism by a sulfate-reducing bacterium, *Desulfatibacillum aliphaticivorans* strain CV2803T. *Appl. Environ. Microbiol.* 71 2458–3467. 10.1128/AEM.71.7.3458-3467.2005 PMC1169040 16000749

[B13] Davidova I. A. Duncan K. E. Choi O. K. Suflita J. M. (2006). *Desulfoglaeba alkanexedens* gen. nov., sp. nov., an n-alkane-degrading, sulfate-reducing bacterium. *Int. J. Syst. Evol. Microbiol.* 56 2737–2742. 10.1099/ijs.0.64398-0 17158970

[B14] Edwards E. A. Grbić-Galić D. (1994). Anaerobic degradation of toluene and o-xylene by methanogenic consortium. *Appl. Environ. Microbiol.* 60 313–322. 8117084 10.1128/aem.60.1.313-322.1994 PMC201305

[B15] Embree M. Nagarajan H. Movahedi N. Chitsaz H. Zengler K. (2014). Single-cell genome and metatranscriptome sequencing reveal metabolic interactions of an alkane-degrading methanogenic community. *ISME J.* 8 757–767. 10.1038/ismej.2013.187 24152715 PMC3960532

[B16] Foght J. (2008). Anaerobic biodegradation of aromatic hydrocarbons: pathways and prospects. *J. Mol. Microbiol. Biotechnol.* 9 93–120. 10.1159/000121324 18685265

[B17] Fowler S. J. Dong X. Sensen C. W. Suflita J. M. Gieg L. M. (2012). Methanogenic toluene metabolism: community structure and intermediates. *Environ. Microbiol.* 14 754–764. 10.1111/j.1462-2920.2011.02631.x 22040260

[B18] Fowler S. J. Gutierrez-Zamora M.-L. Manefield M. Gieg L. M. (2014). Identification of toluene degraders in a methanogenic enrichment culture. *FEMS Microbiol. Ecol.* 89 625–636. 10.1111/1574-6941.12364 24910080

[B19] Gieg L. M. Duncan K. E. Suflita J. M. (2008). Bioenergy production via microbial conversion of residual oil to natural gas. *Appl. Environ. Microbiol.* 74 3022–3029. 10.1128/AEM.00119-08 18378655 PMC2394919

[B20] Gieg L. M. Fowler S. J. Berdugo-Clavijo C. (2014). Syntrophic biodegradation of hydrocarbon contaminants. *Curr. Opin. Biotechnol.* 27 21–29. 10.1016/j.copbio.2013.09.002 24863893

[B21] Gray N. D. Sherry A. Grant R. J. Rowan A. K. Hubert C. R. J. Callbeck C. M. (2011). The quantitative significance of Syntrophaceae and syntrophic partnerships in methanogenic degradation of crude oil alkanes. *Environ. Microbiol.* 13 2957–2975. 10.1111/j.1462-2920.2011.02570.x 21914097 PMC3258425

[B22] Gray N. D. Sherry A. Hubert C. Dolfing J. Head I. M. (2010). “Methanogenic degradation of petroleum hydrocarbons in subsurface environments: remediation, heavy oil formation, and energy recovery,” in *Advances in Applied Microbiology*, eds Allen S. S. Laskin I. Geoffrey M. G. (Cambridge: Academic Press), 137–161. 10.1016/S0065-2164(10)72005-0 20602990

[B23] Grundmann O. Behrends A. Rabus R. Amann J. Halder T. Heider J. (2008). Genes encoding the candidate enzyme for anaerobic activation of n-alkanes in the denitrifying bacterium HxN1. *Environ. Microbiol.* 10 376–385. 10.1111/j.1462-2920.2007.01458.x 17961174

[B24] Heider J. (2007). Adding handles to unhandy substrates: anaerobic hydrocarbon activation mechanisms. *Curr. Opin. Chem. Biol.* 11 188–194. 10.1016/j.cbpa.2007.02.027 17349816

[B25] Jones D. M. Head I. M. Gray N. D. Adams J. J. Rowan A. K. Aitken C. M. (2008). Crude-oil biodegradation via methanogenesis in subsurface petroleum reservoirs. *Nature* 451 176–180. 10.1038/nature06484 18075503

[B26] Kleinsteuber S. Schleinitzm K. M. Vogt C. (2012). Key players and team play: anaerobic microbial communities in hydrocarbon-contaminated aquifers. *Appl. Microbiol. Biotechnol.* 94 851–873. 10.1007/s00253-012-4025-0 22476263

[B27] Liang B. Wang L.-Y. Mbadinga S. M. Liu J.-F. Yang S.-Z. Gu J.-D. (2015). *Anaerolineaceae* and *Methanosaeta* turned to be the dominant microorganisms in alkanes-dependent methanogenic culture after long-term of incubation. *AMB Express* 5 37 10.1186/s13568-015-0117-4 PMC4469597 26080793

[B28] Mathai P. P. Zitomer D. H. Maki J. S. (2015). Quantitative detection of syntrophic fatty acid-degrading bacterial communities in methanogenic environments. *Microbiology* 161 1189–1197. 10.1099/mic.0.000085 25814038

[B29] Mbadinga S. M. Li K.-P. Zhou L. Wang L.-Y. Yang S.-Z. Liu J.-F. (2012). Analysis of alkane-dependent methanogenic community derived from production water of a high-temperature petroleum reservoir. *Appl. Microbiol. Biotechnol.* 96 531–542. 10.1007/s00253-011-3828-8 22249716

[B30] Musat F. (2015). The anaerobic degradation of gaseous, nonmethane alkanes – From in situ processes to microorganisms. *Comput. Struct. Biotechnol. J.* 13 222–228. 10.1016/j.csbj.2015.03.002 25904994 PMC4402382

[B31] Nelson M. C. Morrison M. Yu Z. (2011). A meta-analysis of the microbial diversity observed in anaerobic digesters. *Bioresour. Technol.* 102 3730–3739. 10.1016/j.biortech.2010.11.119 21194932

[B32] Notredame C. Higgins D. G. Heringa J. (2000). T-Coffee: a novel method for fast and accurate multiple sequence alignment. *J. Mol. Biol.* 302 205–217. 10.1006/jmbi.2000.4042 10964570

[B33] Pruesse E. Quast C. Knittel K. Fuchs B. M. Ludwig W. Peplies J. (2007). SILVA: a comprehensive online resource for quality checked and aligned ribosomal RNA sequence data compatible with ARB. *Nucleic Acids Res.* 35 7188–7196. 10.1093/nar/gkm864 17947321 PMC2175337

[B34] Rotaru A. E. Shrestha P. M. Liu F. Markovaite B. Chen S. Nevin K. P. (2014). Direct interspecies electron transfer between Geobacter metallireducens and *Methanosarcina barkeri*. *Appl. Environ. Microbiol.* 80 4599–4605. 10.1128/AEM.00895-14 24837373 PMC4148795

[B35] Saito Y. Aoki M. Hatamoto M. Yamaguchi T. (2015). Presence of a novel methanogenic archaeal lineage in anaerobic digesters inferred from mcrA and 16S rRNA gene phylogenetic analyses. *J. Water Environ. Technol.* 13 279–289. 10.2965/jwet.2015.279

[B36] Savage K. N. Krumholz L. R. Gieg L. M. Parisi V. A. Suflita J. M. Allen J. (2010). Biodegradation of low-molecular-weight alkanes under mesophilic, sulfate-reducing conditions: metabolic intermediates and community patterns. *FEMS Microbiol. Ecol.* 72 485–495. 10.1111/j.1574-6941.2010.00866.x 20402777

[B37] Shannon P. Markiel A. Ozier O. Baliga N. S. Wang J. T. Ramage D. (2003). Cytoscape: a software environment for integrated models of biomolecular interaction networks. *Genome Res.* 13 2498–2504. 10.1101/gr.1239303 14597658 PMC403769

[B38] Sherry A. Gray N. D. Ditchfield A. K. Aitken C. M. Jones D. M. Röling W. F. M. (2013). Anaerobic biodegradation of crude oil under sulphate-reducing conditions leads to only modest enrichment of recognized sulphate-reducing taxa. *Int. Biodeter. Biodegr.* 81 105–113. 10.1016/j.ibiod.2012.04.009

[B39] Sieber J. R. McInerney M. J. Gunsalus R. P. (2012). Genomic insights into syntrophy: the paradigm for anaerobic metabolic cooperation. *Annu. Rev. Microbiol.* 66 429–452. 10.1146/annurev-micro-090110-102844 22803797

[B40] Sikkema J. De Bont J. A. Poolman B. (1995). Mechanisms of membrane toxicity of hydrocarbons. *Microbiol. Rev.* 59 201–222. 7603409 10.1128/mr.59.2.201-222.1995 PMC239360

[B41] Soh J. Dong X. Caffrey S. M. Voordouw G. Sensen C. W. (2013). Phoenix 2: a locally installable large-scale 16S rRNA gene sequence analysis pipeline with Web interface. *J. Biotechnol.* 167 393–403. 10.1016/j.jbiotec.2013.07.004 23871656

[B42] Stadtman T. C. Barker H. A. (1951). Studies on methane fermentation. VIII. Tracer experiments on fatty acid oxidation by methane bacteria. *J. Bacteriol.* 61 67–80. 14824080 10.1128/jb.61.1.67-80.1951 PMC385962

[B43] St-Pierre B. Wright A. D. G. (2013). Comparative metagenomic analysis of bacterial populations in three full-scale mesophilic anaerobic manure digesters. *Environ. Biotechnol.* 98 2709–2717. 10.1007/s00253-013-5220-3 24085391

[B44] Symons G. E. Buswell A. M. (1933). The methane fermentation of carbohydrates. *J. Am. Chem. Soc.* 55 2028–2036. 10.1021/ja01332a039

[B45] Tamura K. Nei M. (1993). Estimation of the number of nucleotide substitutions in the control region of mitochondrial DNA in humans and chimpanzees. *Mol. Biol. Evol.* 10 512–526. 8336541 10.1093/oxfordjournals.molbev.a040023

[B46] Tamura K. Stecher G. Peterson D. Filipski A. Kumar S. (2013). MEGA6: molecular evolutionary genetics analysis version 6.0. *Mol. Biol. Evol.* 30 2725–2729. 10.1093/molbev/mst197 24132122 PMC3840312

[B47] Tan B. Fowler S. J. Abu Laban N. Dong X. Sensen C. W. Foght J. (2015a). Comparative analysis of metagenomes from three methanogenic hydrocarbon-degrading enrichment cultures with 41 environmental samples. *ISME J.* 9 2028–2045. 10.1038/ismej.2015.22 25734684 PMC4542035

[B48] Tan B. Nesbø C. Foght J. (2014). Re-analysis of omics data indicates *Smithella* may degrade alkanes by addition to fumarate under methanogenic conditions. *ISME J.* 8 2353–2356. 10.1038/ismej.2014.87 24865771 PMC4260707

[B49] Tan B. Semple K. Foght J. (2015b). Anaerobic alkane biodegradation by cultures enriched from oil sands tailings ponds involves multiple species capable of fumarate addition. *FEMS Microbiol. Ecol.* 91:fiv042 10.1093/femsec/fiv042 25873461

[B50] Townsend G. T. Prince R. C. Suflita J. M. (2003). Anaerobic oxidation of crude oil hydrocarbons by the resident microorganisms of a contaminated anoxic aquifer. *Environ. Sci. Technol.* 37 5213–5218. 10.1021/es0264495 14655710

[B51] Větrovský T. Baldrian P. (2013). The variability of the 16S rRNA gene in bacterial genomes and its consequences for bacterial community analyses. *PLoS ONE* 8:e57923 10.1371/journal.pone.0057923 PMC3583900 23460914

[B52] von Netzer F. Pilloni G. Kleindienst S. Krüger M. Knittel K. Gründger F. (2013). Enhanced gene detection assays for fumarate-adding enzymes allow uncovering of anaerobic hydrocarbon degraders in terrestrial and marine systems. *Appl. Environ. Microbiol.* 79 543–552. 10.1128/AEM.02362-12 23124238 PMC3553772

[B53] Washer C. E. Edwards E. A. (2007). Identification and expression of benzylsuccinate synthase genes in a toluene-degrading methanogenic consortium. *Appl. Environ. Microbiol.* 73 1367–1369. 10.1128/AEM.01904-06 17142355 PMC1828654

[B54] Widdel F. Musat F. (2010). “Diversity of common principles in enzymatic activation of hydrocarbons,” in *Handbook of Hydrocarbon and Lipid Microbiology*, ed. Timmis K. N. (Berlin: Springer-Verlag), 983–1009.

[B55] Yamada T. Sekiguchi Y. Hanada S. Imachi H. Ohashi A. Harada H. (2006). *Anaerolinea thermolimosa* sp. nov., *Levilinea saccharolytica* gen. nov., sp. nov. and *Leptolinea tardivitalis* gen. nov., sp. nov., novel filamentous anaerobes, and description of the new classes Anaerolineae classis nov. and Caldilineae classis nov. in the bacterium phylum Chloroflexi. *Int. J. Syst. Evol. Microbiol.* 56 1331–1340. 16738111 10.1099/ijs.0.64169-0

[B56] Zengler K. Richnow H. Rosselló-Mora R. Michaelis W. Widdel F. (1999). Methane formation from long-chain alkanes by anaerobic microorganisms. *Nature* 401 266–269. 10.1038/45777 10499582

