# Supplementary material for: Community Structure in Methanogenic Enrichments Provides Insight into Syntrophic Interactions in Hydrocarbon-Impacted Environments
Source: Front Microbiol. 2016 Apr 22;7:562. doi: 10.3389/fmicb.2016.00562 (PMC4840303; doi:10.3389/fmicb.2016.00562)
Supplement: Supplementary file 1 [file Data_Sheet_1.PDF]

SUPPLEMENTAL INFORMATION

‘Community Structure in Methanogenic Enrichments Provides Insight into Syntrophic Interactions in Hydrocarbon-Impacted Environments’

S. J. Fowler, C. R. A. Toth, and L. M. Gieg

1 Supplemental Figure

Figure S1

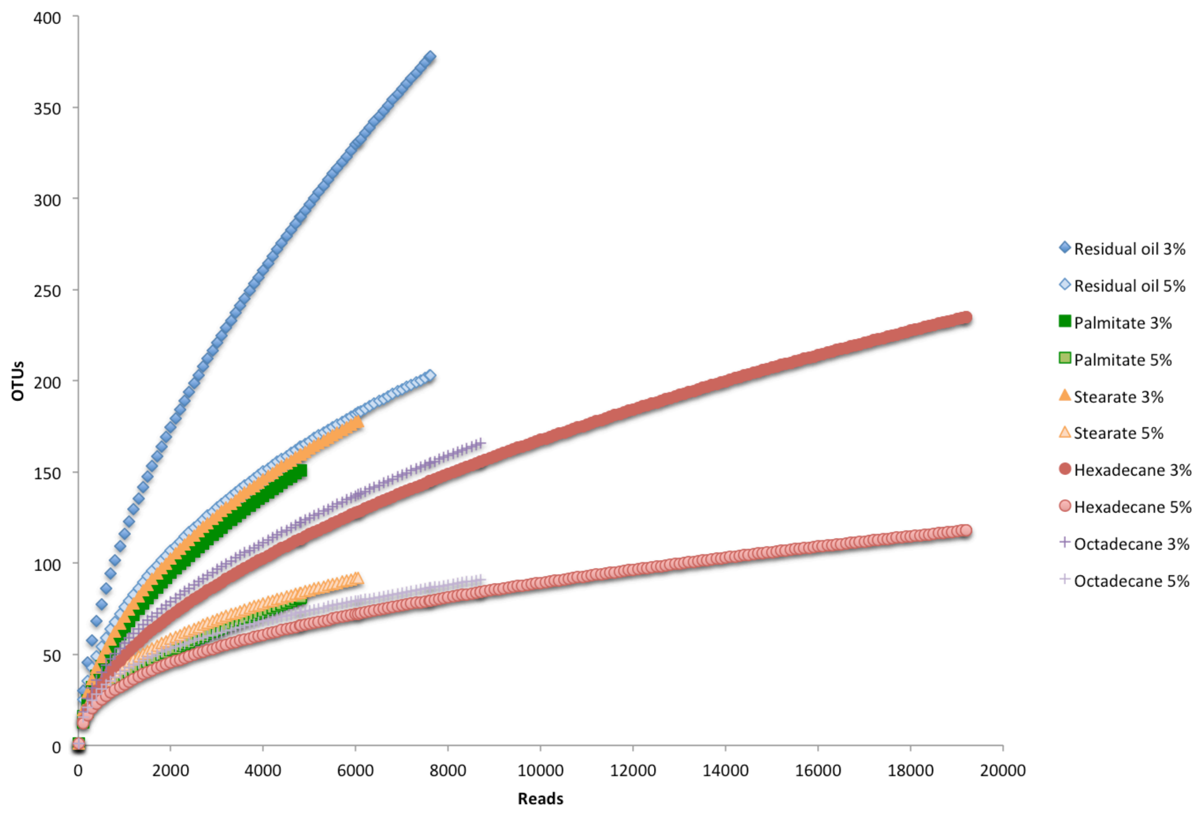

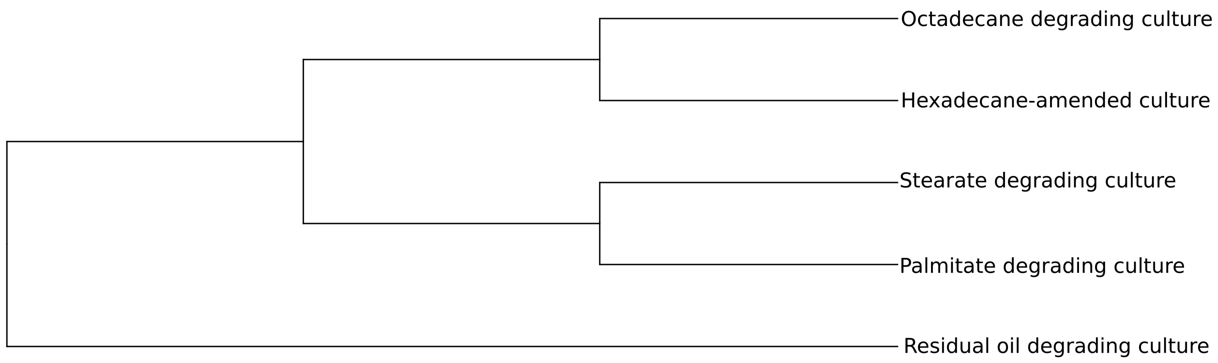

Figure S1. Top: Rarefaction curve results for 16S rRNA gene sequences for methanogenic enrichments sequenced by pyrotag sequencing at 3 and 5% clustering distance. Bottom: Results of Bray-Curtis dissimilarity analysis.
